# A REDCap-based model for online interventional research: Parent sleep education in autism

**DOI:** 10.1017/cts.2021.798

**Published:** 2021-06-14

**Authors:** Beth A. Malow, Anjalee Galion, Frances Lu, Nan Kennedy, Colleen E. Lawrence, Alison Tassone, Lindsay O’Neal, Travis M. Wilson, Robert A. Parker, Paul A. Harris, Ann M. Neumeyer

**Affiliations:** 1 Sleep Disorders Division, Department of Neurology, Vanderbilt University Medical Center, Nashville, TN, USA; 2 Division of Pediatric Neurology, Department of Pediatrics, University of California Irvine – Children’s Hospital of Orange County, Orange, CA, USA; 3 Biostatistics Center, Massachusetts General Hospital, Boston, MA, USA; 4 Vanderbilt Institute for Clinical and Translational Research, Vanderbilt University Medical Center, Nashville, TN, USA; 5 Lurie Center for Autism, Massachusetts General Hospital, Harvard Medical School, Lexington, MA, USA; 6 Department of Biomedical Informatics, Department of Biostatistics, and Department of Biomedical Engineering, Vanderbilt University, Nashville, TN, USA

**Keywords:** Sleep disorder, autism, REDCap, online platform, sleep education

## Abstract

**Introduction:**

The use of online platforms for pediatric healthcare research is timely, given the current pandemic. These platforms facilitate trial efficiency integration including electronic consent, randomization, collection of patient/family survey data, delivery of an intervention, and basic data analysis.

**Methods:**

We created an online digital platform for a multicenter study that delivered an intervention for sleep disorders to parents of children with autism spectrum disorder (ASD). An advisory parent group provided input. Participants were randomized to receive either a sleep education pamphlet only or the sleep education pamphlet plus three quick-tips sheets and two videos that reinforced the material in the pamphlet (multimedia materials). Three measures – Family Inventory of Sleep Habits (FISH), Children’s Sleep Habits Questionnaire modified for ASD (CSHQ-ASD), and Parenting Sense of Competence (PSOC) – were completed before and after 12 weeks of sleep education.

**Results:**

Enrollment exceeded recruitment goals. Trial efficiency was improved, especially in data entry and automatic notification of participants related to survey completion. Most families commented favorably on the study. While study measures did not improve with treatment in either group (pamphlet or multimedia materials), parents reporting an improvement of ≥3 points in the FISH score showed a significantly improved change in the total CSHQ (*P* = 0.038).

**Conclusion:**

Our study demonstrates the feasibility of using online research delivery platforms to support studies in ASD, and more broadly, pediatric clinical and translational research. Online platforms may increase participant inclusion in enrollment and increase convenience and safety for participants and study personnel.

## Introduction

The use of online platforms for healthcare research is gaining interest [1]. In contrast to conventional, in-person methods, online research allows for the collection of data remotely, thereby allowing researchers to take new approaches to their work. A basic example of online research involves emailing a survey. However, online research has applications that go beyond survey collection. An online platform that is well developed and specifically dedicated to clinical and translational research can facilitate the integration of multiple research functions, including recruitment and consent, collection of patient/family survey data, delivery of information, including interventions, and basic data analysis resulting in increased trial efficiency. In addition to these substantial operational advantages for researchers, an online platform can facilitate participant recruitment through increased convenience and safety for participants and staff, improve participant inclusion and enrollment/retention, and contribute to an effective recruitment strategy and outreach. Several trial enhancement options and potential barriers to conduct research studies on digital platforms are listed in Table 1, and we address these further below.

**Table 1.** Enhancement opportunities and challenges to address when considering digital platforms for Clinical and Translational Research

Enhancement opportunities	Challenges to address
Increased participant inclusion and consent/enrollment/retention with wider geographic catchment area and better study access for those who have difficulty participating in person due to disability or caregiving responsibilities	As study personnel are not engaging with participants face to face, a patient-centered approach is critical (involving study population in research design, continuously engaging participants to ensure full study participation through email reminders and phone calls). Because staff may not be immediately available, and a participant may be engaging with the digital platform, instructions for participants must be very concise and clear. Digital inequity might not allow everyone to participate. Participants may need to be loaned electronic devices to fully participate in study.
Increased convenience and safety for participants and study personnel (outside of normal work times, during pandemics)	
Increased innovation and efficiency (data entry by participants directly into the study platform, real-time reporting tools for study personnel, modifications can be rolled out immediately)	Underfunded studies may lack the resources to pay programmers to develop custom modules and retain a digital support team for the duration of a study to ensure that these modules continue to function properly. Leveraging existing supported platforms for most technical needs and paying only for customization or extension when required for study optimization can reduce costs.

### Increased Participant Inclusion and Enrollment/Retention

Online platforms facilitate remote participation and thus can dramatically increase the catchment area for a study. Families can participate from anywhere, including other states. By minimizing geographic barriers, both total enrollment and geographic diversity of the study population can be enhanced [2, 3]. Other recruitment methods (e.g., social media, registries) can be used in a complementary manner to increase reach and overall enrollment [3, 4].

In rural areas without access to a research center, remote platforms may be the only option. Online platforms may also be designed to improve inclusivity in other ways. In cases where the study population is not expected to be monolingual, the platform and data collection instruments can be translated into other languages. By reducing the burden on participant families, well-designed online platforms may increase the sense of connectedness with the study and therefore increase retention rates.

### Increased Convenience and Safety for Participants

Families can participate from home and do not need to come to medical centers that may be far away or difficult to access (e.g., commute, parking, mobility, childcare, and navigating logistics required to bring a child to a research visit). They can participate on their own time at their convenience, within established study time windows. Remote participation also poses less risk and anxiety to research participants and families in terms of contracting or spreading infectious disease, especially during a pandemic (e.g., COVID-19) [5], and in fact, may be the only permissible option for conducting a research study that minimizes face-to-face contact with participants in such a situation. Moreover, online studies can be particularly welcome to families of individuals with disabilities or other special needs that could affect their participation (e.g., increased anxiety about coming to a large medical center for study visits).

### Increased Efficiency

Online platforms are efficient in terms of manpower, costs, and paper needs. Because study coordinators can carry out their work without dedicated clinical space for receiving participants, research conducted using online platforms can greatly increase the capacity to conduct larger studies with less personnel and physical facility requirements than traditional face-to-face research study methodology [2–4, 6, 7]. In models where the patient or family enters data directly into an electronic data capture system, coordinators are relieved of scheduling and data entry tasks that normally require significant dedicated effort [3, 8], with reduced opportunity for error. Efficiency gains in communication between Principal Investigators (PIs) can also be achieved by reporting tools based on up-to-date data related to recruitment and study activities [3]. Modifications in digital platforms can be rolled out immediately to all participants and all study sites, thus greatly reducing the time and expense of logistical planning for non-digital material distribution [9].

### Digital Equality

As already noted, an online platform can widen the access to study participation, enabling many more people to take part who otherwise might face insurmountable time, travel, or emotional burdens. To facilitate maximal inclusion of participants, it is important to optimize in-platform system design so that the expectations for the tasks participants need to complete are clear and concise. Additionally, participation in a study relying on a digital platform may be limited by digital access (devices, Internet) or technical proficiency. Recent reports show that 81% of US adults now own a smartphone, 74% a desktop computer, and 52% a tablet [10]. Notably, smartphone ownership is nearly as high among Black and Hispanic populations as Whites (80% and 79% vs. 82%) and reaches 71% of those with income under $30,000 [10]. Home Internet access has reached the saturation point (99%) [11]. While just 73% say they use broadband [12], close to half (45%) of individuals without home broadband say their smartphone connection fills that void [13]. Hardware considerations may require budgeting for loaner devices so that those few participants without access to computers or smartphones can still complete the study, thereby improving study participation and generalizability. While this may incur a cost, the return of value in terms of incentivizing recruitment may be worthwhile [14].

### Social Connectedness and Mutual Trust

Use of an online platform can lead to novel opportunities to establish trust and strengthen participant engagement. While in-person experiences may allow participants to feel more fully connected [8], a “patient-centered” approach that includes involving the patient or family directly in research functions and obtaining their feedback may enrich engagement and connectedness. Maintaining direct contact between the patient/family and coordinators or clinicians with whom they have an established trust relationship, from the time of phone screening to study completion, can be helpful. Additional engagement and retention strategies include periodic phone calls, electronic messaging, dissemination of study news, and personalized thank you cards sent by mail.

### Digital Trust and Data Privacy Considerations

With online health care becoming more common, new safeguards have been put into place for many of the platforms, which are now being used for remote work, eConsent, and telehealth. Online studies can further protect themselves from inadvertent exposure of protected data by limiting the use of social media platforms for anything other than engagement and recruitment purposes. While there is evidence that public trust in data privacy has become precarious due to several high-profile data breaches and dishonorable data-sharing practices in the online environment, research teams can take proactive steps to clearly articulate, in lay language, the controls, and measures in place to protect inappropriate access to research data [3]. To build and maintain trust, it is also important that research teams clearly explain data-sharing policies and procedures.

### Electronic Consent (eConsent)

The availability of eConsent greatly facilitates online research, as it may reduce barriers for consenting rural populations and increase near- and long-term understanding of a study through the use of non-paper features such as video, comprehension questions, and other “just in time” supplemental content. The eConsent platform can be accessed on a variety of electronic devices including PC, tablets, and smartphones. Various remote consent workflow models have been developed and described [15, 16]. Importantly, research that includes children requires the involvement of legal guardians (e.g., families) to facilitate the consent/assent process [17, 18]. Regardless of the workflow and technical platform, established principles of consenting must be upheld [19]. Different US states and local Institutional review boards have unique requirements for the systems that are used to implement electronic signatures. Therefore, not all states can utilize the eConsent Framework, and sites within those states may need to use paper consents. Additionally, even if a state can use eConsent, a site may wish to consider making paper consent documents available to meet the needs of participants who prefer paper documents.

### Innovation Opportunities

Although unknowns are an unavoidable aspect of innovative technologies, embracing community engagement early in the process of study design and operational planning can reduce uncertainty and increase the probability of success [20]. Custom technology development is expensive and can be prohibitive in small studies, where budgets will not sustain experimentation or iteration. Furthermore, once custom modules to a platform are developed, it is necessary to retain a digital support team throughout the study to ensure that these modules continue to function properly. The risk here can be decreased by leveraging existing, supported platforms for the majority of technical needs [3] and paying only for customization or extension when required for study optimization [21, 22].

## Current Study

We describe here our experience and lessons learned creating an online digital platform in support of a multicenter study, which was carried out within three sites in the Autism Intervention Research Network on Physical Health (AIR-P). The study was designed to benefit the autism community through the improvement of insomnia, defined as difficulty initiating or maintaining sleep. Sleep education, including attention to daytime habits, evening routines, the timing of sleep, and parent interactions with their children, has shown benefit in improving sleep, child behavior, and family functioning [23–28], and practice guidelines have emphasized its use as the first-line treatment [29, 30]. Delivery of sleep education to reach as many families as possible (including those without easy access to practitioners) can be facilitated through apps and other online methods [31]. Our approach included utilization and extension of an existing, well-supported Research Electronic Data Capture tool (REDCap)[32, 33] online platform, which is freely available to academic, nonprofit, and government organizations around the globe and is now supporting more than 1.3M end users across over 4000 institutions in 137 countries. REDCap is conducive in incorporating participant consent, survey data, and interventions, with special features such as tracking of participant responses and accessing interventions. We believe that the framework and approach described in this manuscript have the potential to support similar and additional innovation models in autism and, more broadly, many other aspects of clinical and translational research.

## Methods

### Study Team, Participants, and Inclusion/Exclusion Criteria

Our team consisted of an overall PI and site PIs, lead coordinator and two site coordinators, a regulatory consultant, technical analysts and developers, statisticians, and families who provided important input into study design and the Parent Portal.

Family advisory members from the AIR-P reviewed and provided input on study design prior to implementation. These included two parents of children with autism spectrum disorder (ASD) who were bilingual in Spanish and one parent of a child with ASD who worked as a community pediatrician. Input was provided via group meetings with investigators and coordinators and via email correspondence (e.g., review of Parent Portal).

**Fig. 1.**
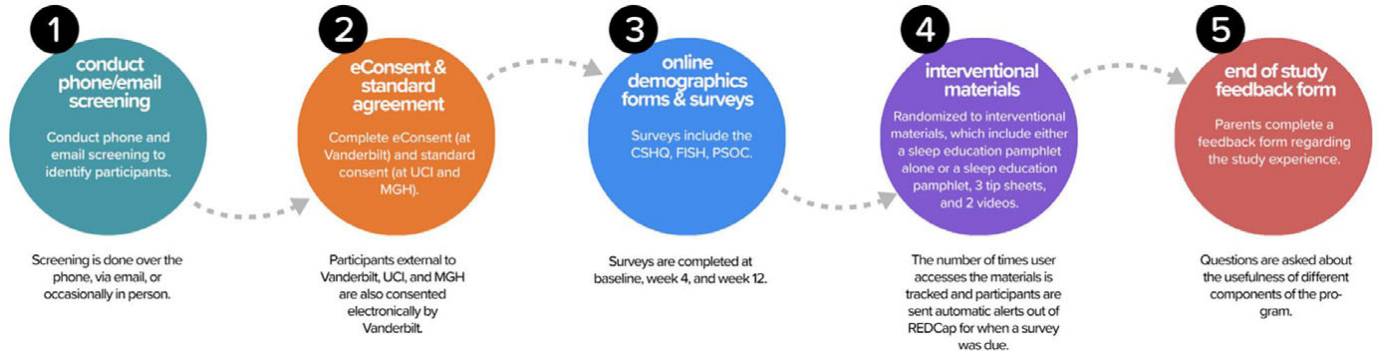
**Study Flow.** After the screening was conducted, participants completed electronic consent at Vanderbilt and standard consent at the other two sites. Online surveys were then completed at baseline, week 4, and week 12. Families were randomized to interventional materials and the number of times the user accessed the materials were tracked. Automated alerts were sent when surveys were due. An end-of-study feedback form was provided to families at the completion of the study. CSHQ, Children’s Sleep Habits Questionnaire; FISH, Family Inventory of Sleep Habits; MGH, Massachusetts General Hospital; PSOC, Parenting Sense of Competence; REDCap, Research Electronic Data Capture; UCI, University of California Irvine.

Parents of children with ASD were enrolled in this study through three different sites: Vanderbilt University Medical Center (VUMC), University of California Irvine (UCI) through the Children’s Hospital of Orange County (CHOC), and the Massachusetts General Hospital (MGH) at the Lurie Center for Autism. Participants were recruited through clinician referrals, study flyers, and social media posts. Referrals were initially contacted by phone, email, or in person (taking into account patient preference), and if referrals were interested in participating, study coordinators provided a brief overview of the study and asked basic eligibility questions (Fig. 1, Circle 1).

Institutional review board approval was received at all sites. All parent participants provided informed consent prior to participating in any study procedures. Parents qualifying for the study at UCI/CHOC, or MGH completed standard consent forms in-person (rather than online eConsent) because these institutions did not yet have eConsent approved. Parents qualifying for the study at VUMC, or external to UCI/CHOC or MGH, completed the consenting process using eConsent, accessed through a REDCap link (Fig. 1, Circle 2). The eConsent allowed for families distant from a study site (including in other states) to participate. Study inclusion criteria included (1) aged 2–10, 11 months; (2) a documented diagnosis of ASD with a validated tool, such as the Autism Diagnostic Observation Schedule (ADOS) [34] or by an appropriate healthcare provider using standardized criteria, such as those defined in the Diagnostic and Statistical Manual (DSM-IV or DSM-5); (3) sleep concerns, including difficulty falling asleep, bedtime resistance, or night wakings; (4) stable medications for at least 2 weeks prior to enrollment in the study and no plans to change or start any new medications throughout the course of the study. English proficiency was required to fill out surveys. Participants were excluded if their child had an untreated medical condition or were anticipated to have changes in psychotropic medications or if they had prior exposure to the sleep education materials.

## Study Design and Intervention

**Fig. 2.**
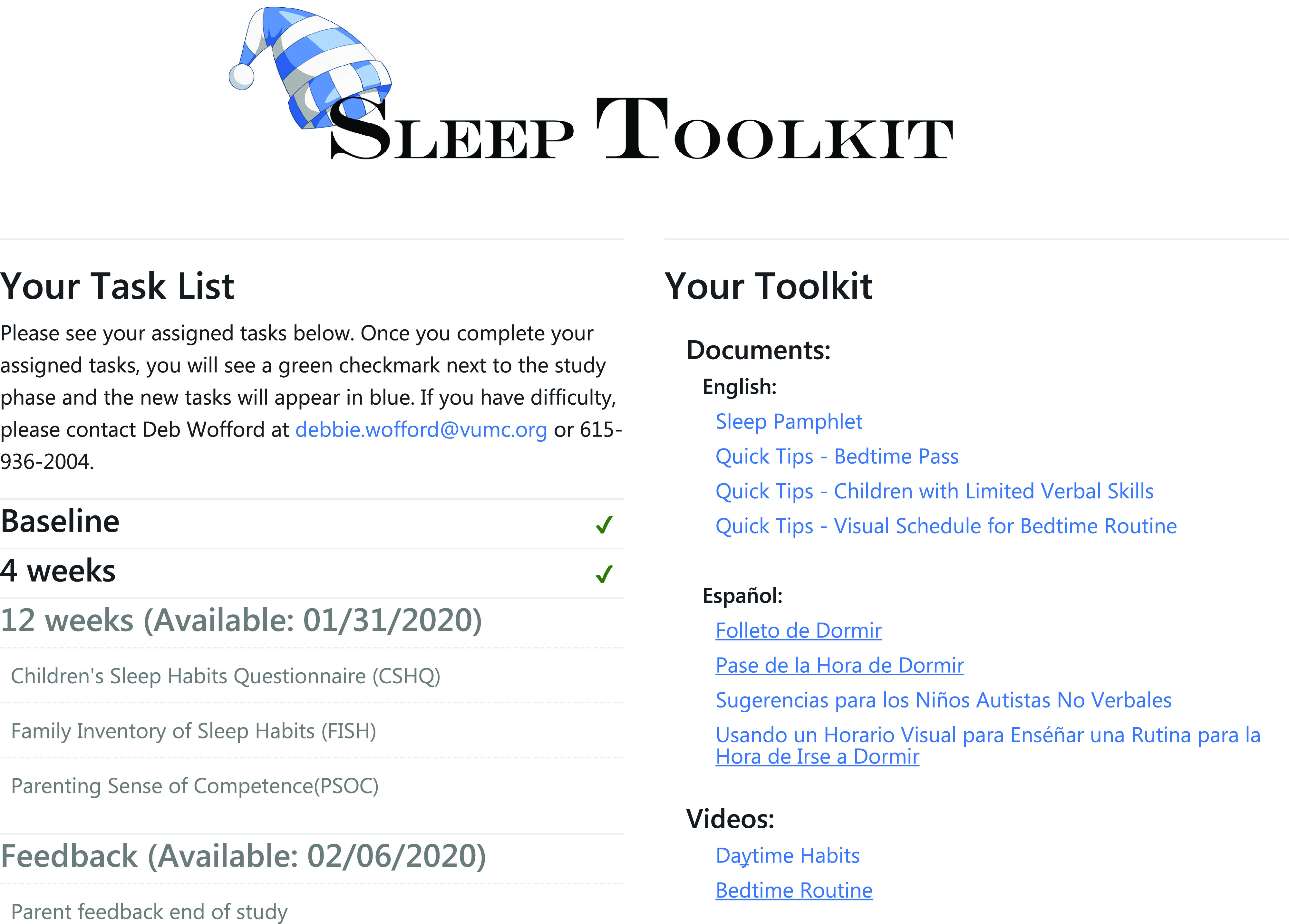
**Parent Portal.** This customized feature allowed for “one-stop shopping” to complete study documents (surveys, end-of-study feedback form) and access interventional materials.

Once the standard consent form or eConsent was signed, the parent was given access to a Parent Portal (Fig. 2), a customized feature designed with feedback from our family advisory members, which allowed for “one-stop shopping” to complete study documents (surveys, end-of-study feedback form) and access interventional materials. The REDCap survey login feature added an additional level of security and verification. In addition to a secure link, the parent was required to enter the child’s date of birth to access the demographic survey at the beginning of the study.

An external module was used to customize the dashboard with the coordinators’ names, emails, and phone numbers for each site. This feature allowed families easy access to their coordinator as questions arose during the study.

## Surveys

Parents first completed a demographic form and baseline surveys (Children’s Sleep Habits Questionnaire modified for ASD – CSHQ-ASD; [35] Family Inventory of Sleep Habits – FISH; [36] Parenting Sense of Competence Scale – PSOC [37]) (Circle 3). These forms and surveys were created using standard REDCap data collection instruments, and included calculations of data fields (e.g., subscales and total scale scores based on survey responses) for statistical analysis. To ensure data integrity, parents were alerted if a field had missing data or out-of-range data. At week 4 and week 12, these surveys were completed again. Parents were sent an automated reminder when it was time to complete these surveys.

The CSHQ-ASD [35] is an abbreviated 23-question, 4-factor version of the caregiver-reported CSHQ [38]. The four factors are Sleep Initiation and Duration, Sleep Anxiety/Co-Sleeping, Night Waking/Parasomnias, and Daytime Alertness (scored on a 3-point scale). These factors make up the subscales of the CSHQ-ASD. A total score can also be calculated. More sleep problems result in higher scores. The CSHQ-ASD showed improvement in a behavioral sleep intervention [39].

The FISH is a 12-item survey of sleep habits (scored on a 5-point scale), including bedtime routine, sleep environment, and parental interactions [36]. A higher score indicates better sleep hygiene. In a behavioral sleep intervention, PSOC scores significantly improved [25]. We considered an increase of 3 or more points in the FISH score to be a clinically meaningful improvement in sleep.

The PSOC is a self-reported 17-item scale (scored on a 6-point scale) developed to assess parents’ self-esteem [37]. Two subscales provide a measure of self-efficacy, indicative of the parent’s sense of his/her own problem-solving ability and capability as a parent, and a measure of satisfaction with parenting that reflects frustration, anxiety, and motivation with the parenting role [6]. A total score can also be calculated. Higher sense of competence results in higher scores. In a behavioral sleep intervention, PSOC scores significantly improved [5].

After these baseline forms were completed, parents were randomized to receive either (a) a sleep education pamphlet or (b) the sleep education pamphlet, three quick tip sheets, and two videos that reinforced the pamphlet material (Fig. 1, Circle 4). Since we did not need to use an outside randomization tool, the participant received immediate access to the materials for their assigned intervention via the online portal. If parents did not have access to a printer, they were given the option to have the educational materials mailed. The sleep education pamphlet is eight pages in length and contains information about daytime and evening habits, the timing of sleep, bedtime routine, and parent interactions, with information and visuals incorporated to help the parents support and improve their child’s healthy sleep habits. The quick tips sheets provide more detail about using a Visual Schedule, a Bedtime Pass, and Tips for Children with Minimal Verbal Skills. The videos discuss healthy daytime habits and show how to create a bedtime routine with a visual schedule, while showing examples of productive parent/child interactions. Written materials were available in both English and Spanish to meet the needs of bilingual families who preferred to receive education in Spanish. Videos were only available in English. All educational material is currently available to the public at no charge on the Autism Speaks website (www.autismspeaks.org/tool-kit/atnair-p-strategies-improve-sleep-children-autism).

We offered access to a tablet in the event that parents did not have access to computers or smartphones (this turned out not to be needed as every participant had access). Parents without access to a printer were given the option of having the educational materials mailed.

After completion of the week 12 surveys, parents rated the usefulness of the sleep materials (Fig. 1, Circle 5). Questions included how useful the sleep materials were for themselves and their child and how likely they were to use the information from the pamphlet in working on sleep with their child. The rating scale for these questions was from 1 to 5, with 5 being the most useful, most likely to use, and most easily used. They were also encouraged to provide open-ended comments.

Parents were compensated for their time with electronic payments or gift cards and were given access to all materials after completing the study.

## Coordinator Portal

**Fig. 3.**
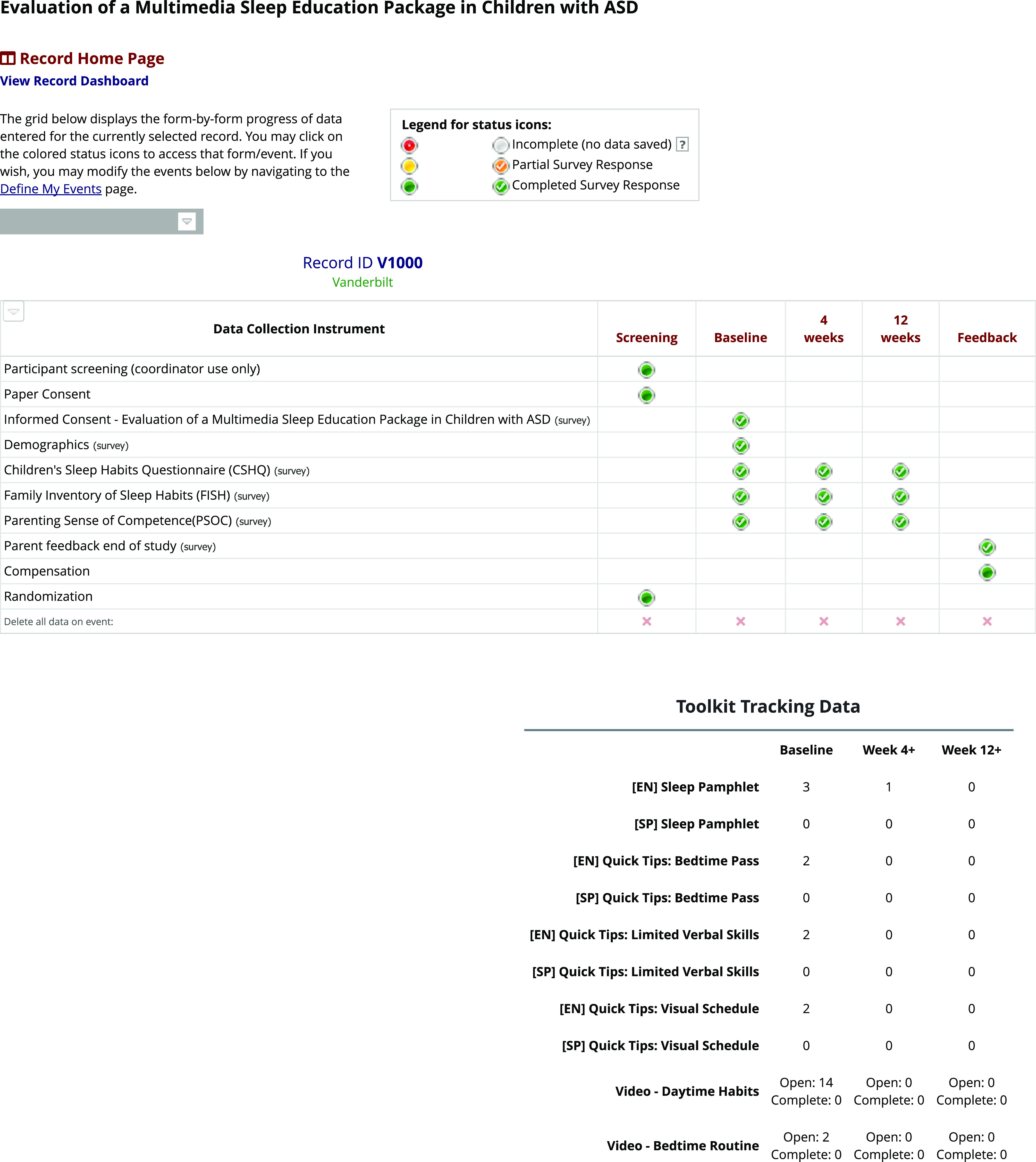
**Data Collection Instruments and Timeline of Activities.** The grid illustrates the data collection instruments and associated study processes (e.g., randomization) to show when each instrument or process was completed. The toolkit tracking tool provided information on which materials were being accessed. ASD, autism spectrum disorder.

Figure 3 illustrates the coordinator view of a single participant. In addition to showing the status of all study activities and questionnaires, the number of times parents accessed the interventional materials were automatically tracked. The lead coordinator received an email notification when baseline, week 4, week 12, and parent feedback were completed, eliminating the need to track completions manually. The lead coordinator communicated this information to the other site coordinators. Coordinators reached out to families initially by email and then by phone if needed when surveys were past due. At one site, upon completion, participants were mailed a “Research Hero” certificate thanking them for their participation.

## Data Analysis

Analyses were completed using SAS 9.4 (SAS Institute Inc, Cary, NC, USA). Demographics and baseline score differences between treatment groups were assessed, stratified by site, using van Elteren’s test for continuous variables and Cochran-Manetel-Haenszel statistics. Demographic and baseline score differences between sites were also assessed using Kruskal–Wallis test and Fisher’s exact test. Viewing of materials by week 4, study completion, and proportion of visits occurring during the COVID-19 pandemic were compared by treatment group. For each assessment score, the effect of treatment at both follow-up time points was modeled using a linear mixed model accounting for repeated measurements within a participant. Fixed effects included treatment group, follow-up time point, viewing of pamphlet materials, school closure at the time point for COVID-19, an interaction term for treatment group by time point, and the baseline score. Site was a random effect. The treatment group effect and the interaction term of treatment by time point were assessed for significance (*P* < 0.05), after adjustment for multiple comparisons using a Holm–Bonferroni adjustment. A *post hoc* analysis was done to assess the impact of treatment adoption using the view counts of media materials, overall and by type of material, as additional fixed effects in the analysis. An additional *post hoc* analysis explored changes in the CSHQ-ASD total score among participants with and without an improvement in the FISH total score.

## Results

### Online Study Design and REDCap Tools

Several features related to the online study design and REDCap tools are highlighted here, including enrollment, randomization, retention, survey completion, and efficiency. Data on child sleep outcomes and learning outcomes in parents of children are also presented.

#### Enrollment

We enrolled a total of 154 participants (median age of 6.6 years; interquartile range of 4.2–8.5; 80% male; 62% White, 9.4% Black, 6.5% Asian, and 36% Hispanic) over a narrow window of time (September 2019–January 2020). This number exceeded our recruitment goal of 125 participants. While most participants were connected with one of the three participating medical centers (VUMC, UCI, or MGH), additional families were able to participate due to the online nature of the study. These included 13 outside of Tennessee, 6 of whom learned about the study through ResearchMatch [40] (FL, OH, WI, MO, NY, and MI).

#### Randomization

Using the REDCap randomization module allowed us to randomize participants (n = 140) once they completed the baseline surveys. After reviewing the data and resolving all inconsistencies, 2 (two) participants were found ineligible, due to an age discrepancy and incomplete baseline surveys. Thus, results for only 138 participants are presented.

#### Survey completion (Retention)

Of 154 participants enrolled, 138 completed baseline surveys, 109 completed week 4 surveys, and 108 completed week 12 surveys. Site study completion rates (completing both surveys at the appropriate time) ranged from 56% to 79%.

#### Efficiency

Labor savings were noted in several areas by our coordinators. The largest impact was on data entry – participants entered data directly into the REDCap portal, minimizing the need for study coordinators to perform data entry. Other areas of labor savings were related to parent notification via the portal when their surveys were ready for completion, and automated tracking of participants to determine who had completed their surveys on time (so that an email or phone call only needed to be sent to remind participants who had not completed surveys).

#### Participant access to online materials

All participants had access to computers or smartphones. Therefore, it was not necessary to provide loaner electronic devices. Ten participants, all at the CHOC site, indicated that they did not have access to a printer. In these cases, parents were either mailed materials or they were sent home with them from their in-person consent appointments. These included five bilingual parents who received Spanish materials.

#### Viewing of materials

Of 138 parents who completed baseline surveys and were eligible to access the educational materials, 114 viewed the materials at least once by week 4 (56 in the pamphlet group and 58 in the multimedia group). Of note, 24 parents had no views of the material (11 in the pamphlet group and 13 in the multimedia group).

#### Sleep data

For the overall treatment effect, change in total FISH (*P* = 0.36) and CSHQ-ASD (*P* = 0.33) did not differ between the pamphlet and all materials groups. The overall treatment effect for the change in PSOC (*P* = 0.03, unadjusted; *P* = 0.09, adjusted for multiple tests) suggested improvement in the all materials group when compared to the pamphlet group, although this was not significant after adjustment for multiple testing. Additional tables of the scores at each time point, overall, and by treatment group are in Supplementary Tables 1 and 2.

Combining both treatment groups, the total CSHQ-ASD score was significantly lower (better) in those with an improved FISH score when compared to those without an improved FISH score (*P* = 0.038; Wilcoxon two-sample test; Table 2). Additionally, results were statistically significant (*P* = 0.019) when limiting the analysis to the group of participants who viewed any materials.

**Table 2.** Change in CSHQ-ASD total score from baseline to week 12, combining participants from the pamphlet and multimedia groups.

FISH improvement by 3 or more points	CSHQ-ASD total score change from baseline to week 12 Median (IQR)	Wilcoxon two-sample test P-value
Yes	−5 (−9, −2)	*P* = 0.038
No	−4 (−7, 0)	

CSHQ-ASD, Children’s Sleep Habits Questionnaire modified for autism spectrum disorder (ASD); FISH, Family Inventory of Sleep Habits.

#### Survey completion during COVID-19

We defined school closures as occurring on or after March 19, 2020. Five participants completed week 4 surveys and 49 participants completed week 12 surveys on or after the date of school closures. There were no significant differences in the proportion of surveys completed after school closures between the treatment groups at week 4 (*P* = 0.61) or week 12 (*P* = 0.40).

#### Feedback from families

End-of-study parent feedback surveys were completed by 64 parents, who rated the usefulness of the study materials and provided open-ended comments. The majority reported that the materials were either useful or extremely useful and likely to use the materials (Table 3). Most had favorable comments, although limitations were noted (Table 4). No technical difficulties were reported during the study.

**Table 3.** Parent ratings at end of study

Question	Pamphlet only (n = 31)	All materials (n = 33)	
Usefulness of materials		Pamphlet and quick tips	Videos
Extremely useful	(7) 23%	(7) 21%	(5) 15%
Useful	(11) 35%	(14) 42.5%	(14) 42.5%
Slightly useful	(11) 35%	(11) 33.5%	(13) 39.5%
Not useful	(2) 7%	(1) 3%	(1) 3%
Likeliness to use materials			
Very likely	(14) 45%	(16) 48.5%	(15) 45.5%
Somewhat likely	(14) 45%	(15) 45%	(14) 42.5%
Somewhat unlikely	(1) 3%	(1) 3%	(4) 12%
Not likely	(2) 7%	(1) 3%	(0) 0%

**Table 4.** Selected parent comments

“This was an amazing opportunity to improve my child’s sleeping problems and I was able to get many options of techniques to support me on this effective way working with my child’s disability and to improve all the bad habits that we just have. Thank you for all of your hard work.”
“It was so easy to go online and do it when I had free time. Thank you for having us participate in your study. It was interesting.”
“I enjoyed this study. I thought the format (all online) was very convenient and the material was straightforward and understandable.”
“Easily accessible, understandable, and implementable information that I found quite helpful for my child’s sleep.”
“Great information, glad that I got both mediums. The video was great to watch, but the paper was good to re-reference.”
“I am very thankful for the opportunity to participate in this study. It did help some with my son’s sleeping habits. The pamphlet was useful, but a little more guidance from a professional during the study would have been more beneficial, I think.”
“I was already doing most of the things, I was looking for something new and novel to help.”
“Pretty standard sleep hygiene materials, so not a lot of new information.”

## Discussion

In this project, we demonstrated the use of online educational interventions for parents of children with ASD. While others have previously reported on online education for children with ASD and sleep problems [41], and found comparable results to face-to-face education, comparisons of two online approaches (pamphlet alone and multimedia materials) to sleep education have not been previously reported to our knowledge. Furthermore, our work illustrates how a research study can be conducted largely online. The use of online software specifically designed for capturing research data and incorporating related research requirements can be instrumental to a study’s success. With the REDCap platform, we were able to conduct remote consent (at one site), deliver the study’s educational intervention, collect survey data, message participants, and track responses, with all these elements folded into a customized Parent Portal dashboard that was designed to sit atop an already established platform – REDCap. Because many REDCap features are “plug and play,” software development expenses were minimized, enabling the use of budgeted funds for customization. REDCap is free to research institutions and offers substantial online training and support to investigators in the use of many elements and features, all of which are highly customizable. Therefore, most of the elements used in this study are available to REDCap users at present.

We did not find that having access to multimedia materials improved sleep when compared to a pamphlet alone. While we saw improvements in sleep habits, sleep patterns, and PSOC after parents received access to the online educational intervention, these improvements were not statistically significant. This may have been due to a variety of factors, including a relatively small sample size coupled with heterogeneity in our study sample (some parents needed more support than an online set of materials could provide). It is important to note that a sizable proportion of parents did not choose to view the online materials. We did find that sleep patterns, as measured by the CSHQ-ASD, improved significantly more in families that had a 3 or more point improvement in FISH score, supporting that improvements in sleep habits are associated with improvements in sleep patterns.

Moreover, other study costs, including study coordinator time, were reduced because online technology obviates the need for in-clinic visits, mailing surveys to parents, and entering data manually. Families remarked that the study procedures were easy and convenient. Another advantage was that the study was able to continue during COVID-19, at a time when many other clinical research studies needed to pause in-person visits due to the risks of participants contracting or spreading infection.

Enrollment exceeded goals. Survey completion (retention) rates were fair. At two sites, phone calls were made to participants who did not respond to email messages regarding overdue surveys. At one site, participants received a completion certificate. We may have improved retention by making phone calls and providing completion certificates to all participants.

Most families appeared satisfied with the process and found the materials useful or extremely useful and were very likely or somewhat likely to use the materials. We believe that engaging the AIR-P family advisory members in the design of the Parent Portal and development of the project, including decisions to include bilingual materials, contributed to the success of this project. Their feedback from a parent perspective was invaluable.

We encountered several participants randomized to the pamphlet-only group who expected to have access to videos. While given access at end of study to all materials, they may have misunderstood that they would have access earlier on in the study. Clearer communication in the consenting process with reinforcement by study coordinators may have been helpful for clarification. Another study limitation is that we did not know how often families viewed materials (families could view printed materials at a later date) so we were not able to precisely measure the “dose” of the intervention. One way to get around this limitation would be to ask families to log the number of times they viewed materials, although this would create an additional family burden.

We made significant efforts to address challenges inherent in the virtual research process, including the potential for digital inequity, loss of privacy, and low engagement; the need for remote consent; and the uncertainties of new technologies. None of our families required tablets, although we purchased several in anticipation of that need. We mailed educational materials to families without access to printers. Each of these challenges was ultimately less problematic than anticipated – mainly because others in the research ecosystem had already faced and addressed them – or because we ourselves found innovative solutions to alleviate them.

## Conclusion

We successfully deployed an existing, well-supported electronic data capture platform to design and conduct research to evaluate an online multimedia sleep education program for parents of children with autism. The platform – REDCap – allowed us to test the effectiveness of our education program in a way that was cost-efficient and highly convenient for families, who were able to participate remotely. Our study was able to be conducted quickly and efficiently, and the processes described are transferable to other studies. This work is especially timely given that it was conducted in part during the COVID-19 pandemic. The research project was able to be continued safely as face-to-face visits were not required. We believe the innovative approach we developed can be adapted by other researchers to study similar online intervention or education delivery programs.

## Supplementary material

For supplementary material accompanying this paper visit https://doi.org/10.1017/cts.2021.798. click here to view supplementary material
