# Supplementary material for: A REDCap-based model for online interventional research: Parent sleep education in autism
Source: J Clin Transl Sci. 2021 Jun 14;5(1):e138. doi: 10.1017/cts.2021.798 (PMC8327546; doi:10.1017/cts.2021.798)
Supplement: Supplementary file 1 [file S2059866121007986sup001.docx]

|  | Timepoint | | |
| --- | --- | --- | --- |
|  | Baseline | Week 4 | Week 12 |
|  | Mean (SD) | Mean (SD) | Mean (SD) |
| CSHQ-ASD Total Score | 44.2 (6.6) | 41.3 (7.2) | 39.8 (7.2) |
| FISH Total Score | 44.3 (6.4) | 45.7 (6.8) | 46.2 (6.2) |
| PSOC Total Score | 72.9 (11.1) | 75.3 (10.7) | 75.3 (10.7) |

Supplemental Table 1. Scores for Both Treatment Groups Combined at Each Timepoint

CSHQ-ASD = Children’s Sleep Habits Questionnaire modified for ASD; FISH = Family Inventory of Sleep Habits; PSOC = Parenting Sense of Competence

|  |  | Pamphlet N=67 | Pamphlet, Videos, Tool Kits N=71 | Test of Overall Treatment Effect |
| --- | --- | --- | --- | --- |
|  |  | Mean (SD) | Mean (SD) | P-value, unadjusted |
| CSHQ-ASD Total Score | Baseline | 44.8 (6.6) | 43.8 (6.6) | 0.33 |
|  | Week 4 | 42.1 (7.0) | 40.4 (7.5) |  |
|  | Week 12 | 40.5 (7.6) | 39.1 (6.6) |  |
| FISH Total Score | Baseline | 44.1 (6.1) | 44.5 (6.8) | 0.36 |
|  | Week 4 | 45.9 (6.3) | 45.6 (7.3) |  |
|  | Week 12 | 46.2 (5.9) | 46.2 (6.5) |  |
| PSOC Total Score | Baseline | 72.6 (10.1) | 73.1 (12.0) | 0.03 |
|  | Week 4 | 75.8 (10.3) | 74.9 (11.2) |  |
|  | Week 12 | 77.8 (11.2) | 74.4 (12.5) |  |

Supplemental Table 2. Scores by Treatment Group at Each Timepoint

CSHQ-ASD = Children’s Sleep Habits Questionnaire modified for ASD; FISH = Family Inventory of Sleep Habits; PSOC = Parenting Sense of Competence
